# The first human experience of a contact force sensing catheter for epicardial ablation of ventricular tachycardia

**DOI:** 10.1007/s12471-014-0554-8

**Published:** 2014-04-08

**Authors:** L. Dabiri Abkenari, F. Akca, N. M. Van Mieghem, T. Szili-Torok

**Affiliations:** 1 Clinical Electrophysiology, Department of Cardiology, Erasmus MC, Rotterdam, the Netherlands; 2 Interventional Cardiology, Department of Cardiology, Erasmus MC, Rotterdam, the Netherlands; 3 Thoraxcenter, Department of Clinical Electrophysiology, Erasmus MC, PO Box 2040, 3000 CA Rotterdam, the Netherlands

**Keywords:** Contact force sensing, Epicardial ablation, Ventricular tachycardia, Radiofrequency ablation

## Abstract

Contact force (CF) is one of the major determinants for sufficient lesion formation. CF-guided procedures are associated with enhanced lesion formation and procedural success. We report our initial experience in epicardial ventricular tachycardia (VT) ablation with a force-sensing catheter using a new approach with an angioplasty balloon. Two patients with arrhythmogenic right ventricular cardiomyopathy who underwent prior unsuccessful endocardial ablation were treated with epicardial VT ablation. CF data were used to titrate force, power and ablation time.

## Introduction

An epicardial approach for ablation of ventricular tachycardia (VT) is often required, since the substrate cannot always be reached entirely from the endocardium [1–3]. Particularly in patients diagnosed with arrhythmogenic right ventricular dysplasia (ARVC) an epicardial ablation approach is frequently inevitable [4]. Radiofrequency (RF) catheter ablation in the epicardial space is substantially different from endocardial ablation. First of all, there is the absence of circulating blood in the epicardium and therefore lack of convective cooling during ablation, the catheter orientation is usually different, and the varying presence of epicardial adipose tissue interferes with lesion formation [5, 6]. These factors all have significant influence on RF lesion formation and should be taken into account during epicardial VT ablation. A European study demonstrated that the overall success rate of epicardial ablation for different aetiologies is 71.6 % [7]. However, during follow-up a significant proportion of 31.4 % experienced recurrence of the tachycardia. In the past, several techniques were developed to improve energy delivery in the epicardial space. Cooled-tip ablation proved to be superior to standard ablation and bipolar ablation resulted in more effective energy delivery than unipolar ablation [8, 9]. In order to further increase the efficacy of epicardial ablation, CF-sensing catheters may play a role.

Contact force (CF) is a very important determinant of lesion formation [10, 11]. Since effective lesion formation in the epicardium can be challenging, we hypothesised that the use of a CF-sensing catheter might contribute to efficacy and safety during epicardial RF ablation. The aim of this paper is to report our initial experience and demonstrate the CF-guided epicardial approach and its feasibility for epicardial VT ablation. This report describes two patients with ARVC who had undergone prior unsuccessful endocardial ablation and were referred for epicardial ablation of VT.

## Methods

Before the procedure, the patients were informed about the epicardial approach and written informed consent was obtained from both patients prior to the procedure. The procedures were performed under general anaesthesia with a cardiothoracic surgical team on standby during the entire intervention. After the procedure a pericardial drain (PeriVac pericardial tray) remained in place for 8–12 h if no significant bleeding or fluid drainage occurred.

### Epicardial access

**Fig. 1:**
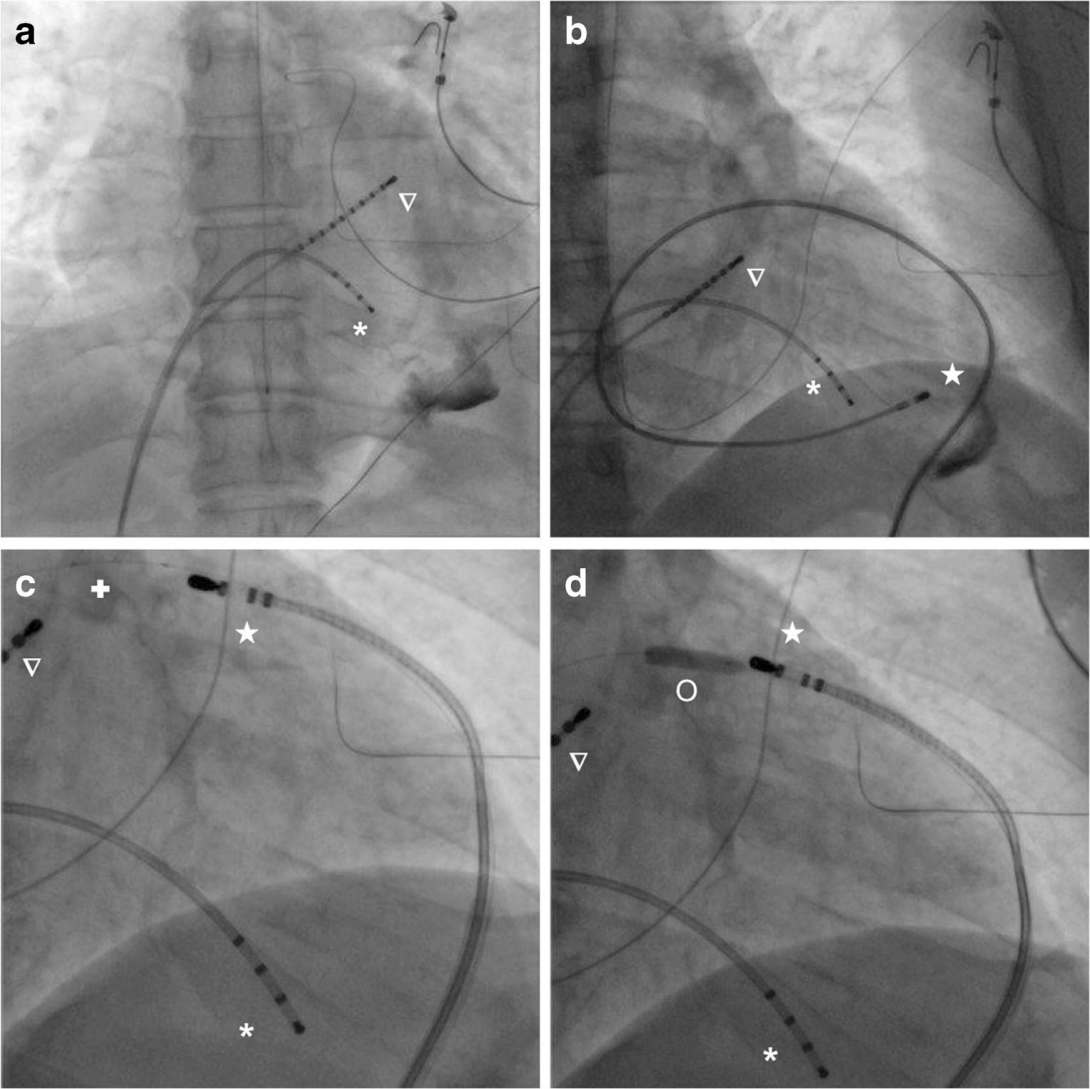
Fluoroscopic recordings during the ablation procedure. **a** Catheter placed in the right ventricle and coronary sinus with contrast fluid visible in the pericardial space. **b** Through the soft flexible vascular sheath, the CF sensing ablation catheter is inserted into the pericardium. **c** Introduction of an additional catheter through the same sheath from the CF catheter. **d** Introduction of the angioplasty balloon in the pericardial space. * Quadripolar catheter into the RV apex. ∇ diagnostic catheter placed in the coronary sinus. ★ CF sensing ablation catheter. ✚ Additional catheter into the pericardium. O Angioplasty balloon advanced into the pericardium

Percutaneous epicardial access was obtained from a subxyphoid level. This was performed with a Tuohy needle (18G, length 15 cm) and a 9 Fr, 24 cm Arrow flex sheath. The pericardial puncture was fluoroscopy guided with the X-ray C arm at 90° latero-lateral projection. Continuous, intermittent small amounts of iodine-based contrast were injected, until the typical layering of the epicardial space was seen (Fig. 1a) [12]. A guide wire (Cordis Exchange wire 35”) was introduced via the needle. Afterwards the soft flexible vascular sheath (9 Fr) was inserted using the Seldinger technique to manipulate the ablation catheter in the pericardium (Fig. 1b).

### CF-sensing catheter

An open irrigated-tip RF catheter with CF sensing technology (TactiCath®, Endosense SA, Geneva, Switzerland) was used. This catheter integrates a CF sensor at the distal part of an RF open-irrigated catheter between the second and third electrode [13]. The force sensor has a deformable body and makes use of infrared laser light and three optical fibres (diameter of 0.125 mm) to detect deformations, which are related to the amount of force applied to the catheter tip [10]. The sensor is able to measure the lateral and axial forces distinctly. The system displays both magnitude and angle of the CF vector with intervals of 100 ms on a separate screen during the ablation procedure. The amount of CF is expressed in grams, and the integrated software calculates a Force-Time Integral^™^ (FTI, function of force and time, expressed in g) [11].

### Mapping and ablation

Both groins were prepared for percutaneous punctures. The chest and abdomen around the xyphoid process were prepared and isolated. A 6 Fr sheath was inserted to gain right femoral venous access for standard diagnostic EP catheters. A quadripolar electrode was placed into the right ventricular (RV) apex to serve as a reference for the electroanatomical mapping system (EAM). Three-dimensional EAM was performed using the NavX^™^ Velocity system (St Jude, St Paul, MN, USA). Endocardial and epicardial bipolar voltage maps were made. Scar tissue was defined as local bipolar electrograms <0.5 mV. Low voltages associated with high-frequency late components were considered as scar area and this was used to create a voltage map. A selective coronary angiogram was performed before power delivery to the epicardial tissue in order to evaluate the proximity to the coronary arteries.

### Calibration of CF catheter

For precise CF measurement, calibration in a no-contact position is required. This was achieved by introduction of an additional catheter together with an angioplasty balloon (5x15 mm compliant Trek coronary dilatation catheter^™^, Abbott Vascular, Santa Clara, USA) through the same sheath (Fig. 1c). The CF catheter is 7 F sheath compatible, making simultaneous introduction of the two catheters possible in the 9 F sheath. The balloon was inflated at 10 Atm just beyond the tip of the catheter in the pericardium (Fig. 1d). Calibration was performed in a single position where the inflated balloon and the ablation catheter tip could be adjacent in a linear fashion. During inflation, the calibration was carried out and the balloon was removed for the rest of the procedure. Dynamic CF data were used to guide the manipulation of the catheter for mapping and RF delivery epicardially.

## Case presentation

### Case 1

**Fig. 2:**
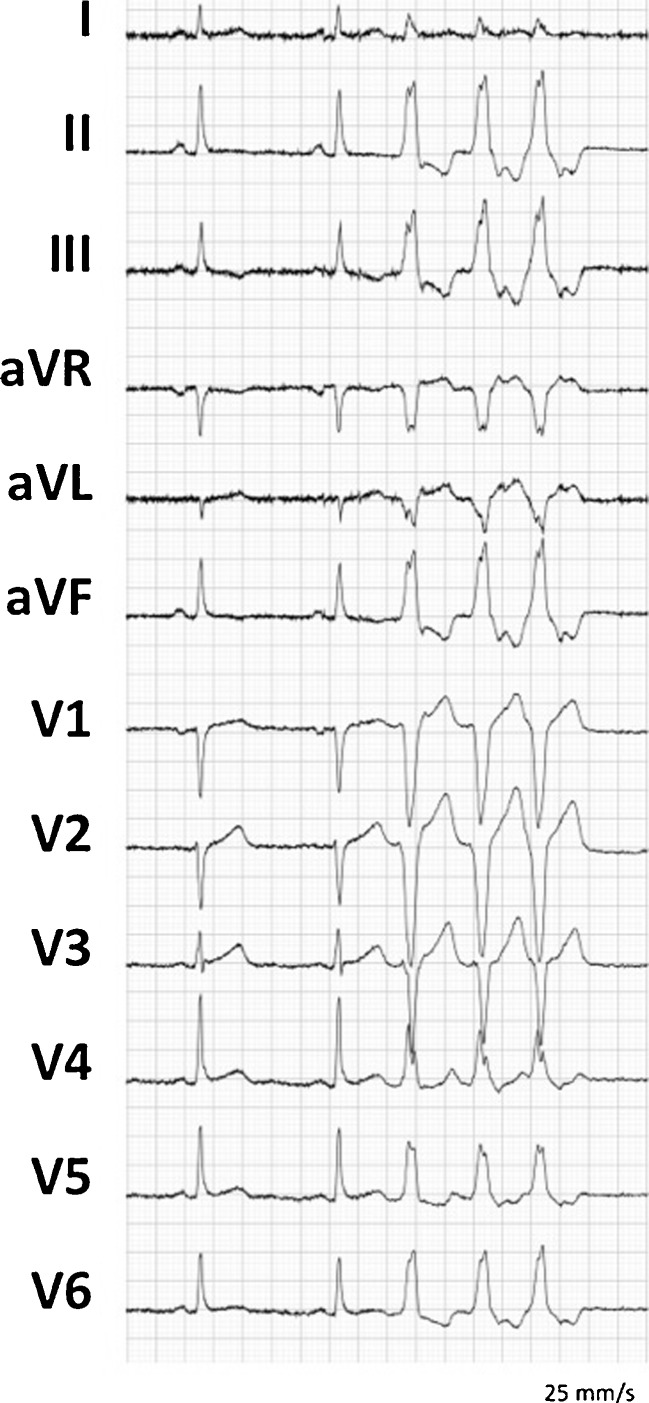
12-lead ECG displaying non-sustained VT with a left bundle branch block morphology and inferior axis, suggestive for RVOT origin

A 67-year-old man diagnosed with ARVC and a preserved systolic left ventricular function presented with multiple episodes of very symptomatic non-sustained VT, which was refractory to medical therapy (Fig. 2a). The VTs had a typical morphology indicating an origin from the right outflow region. The patient had a single chamber defibrillator. Endocardial ablation had been attempted twice (1 year and 3 years previously) and failed to eliminate the substrate of the VTs. An epicardial origin was suspected on the basis of mapping during the endocardial procedure.

During mapping, extensive anterior right ventricular outflow tract (RVOT) epicardial scarring was identified. Other RV regions had no low bipolar voltages. Scar homogenisation was performed endocardially as well as in the adjacent epicardial sites. This led to complete diminution of the premature ventricular contractions, and non-inducibility of VTs during a 30-min waiting period.

The CF remained under 25 g throughout the whole epicardial mapping procedure. In some regions it was very challenging to increase the contact force above 2 g. A total number of 6 RF applications (power: 30–45 W, time: 60 s, irrigation flow >17 mL/min) were applied epicardially. The average force during the ablations was 9 g, with an average FTI of 418 g. In total 33 min of fluoroscopy were used. No audible steam pop was observed during applications. No complications occurred.

After 1 year of follow-up the patient was free from symptoms and ICD interrogation detected no more ventricular arrhythmias. No antiarrhythmic drugs were used during follow-up.

### Case 2

A 48-year-old man, previously a top level sporter, was referred because of incessant VT. Sinus rhythm was obtained by electrical cardioversion. Interestingly enough he had previously undergone RF ablation for idiopathic RVOT tachycardia. The first episode had occurred 16 years ago. He was initially treated with beta-blocker therapy, and remained asymptomatic for 16 years without medication until he presented with haemodynamically stable VTs. After further investigations he was diagnosed with ARVC. This clinical VT was basically different than the one described 16 years ago.

**Fig. 3:**
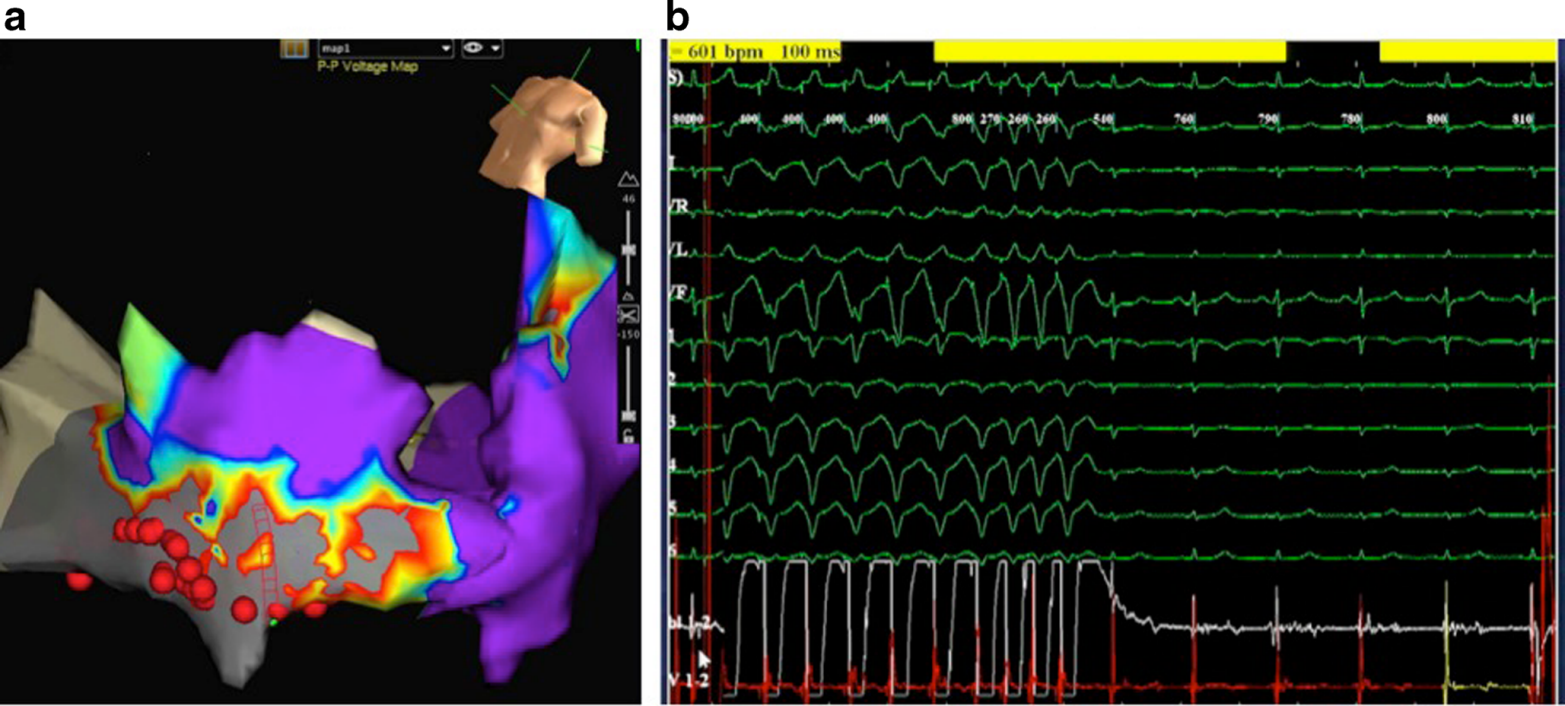
**a** Electroanatomical map from Case 2 after ablation. Sites where RF applications were applied are indicated by red dots. Ablation was performed along the borders of the scar region (marked by grey coloration). **b** Intra-cardiac electrograms at the successful ablation site showing late potentials (fragmented potentials occurring after the end of surface QRS complex) recorded by the ablation catheter. During ablation termination of the tachycardia occurred

During the procedure two distinct VTs (cycle length 370 ms and 330 ms) with left bundle branch block morphology, left axis and initial slurring in the QRS complex were reproducibly induced by programmed ventricular stimulation. Epicardial mapping identified their origin in the apical region of the right ventricle, where dense scar bordered healthy tissue. A linear epicardial line of ablation was drawn from the border of the scar to the healthy tissue (Fig. 3a). The VT terminated during the third ablation point (Fig. 3b). The tachycardia was no longer inducible after ablation, even after a 30-min waiting period.

The average CF was 6 g, and remained under 17 g during the entire mapping procedure. A total number of 14 RF applications (power: 30–45 W, time: 60 s, irrigation flow: 20 ml/min) were applied epicardially. The average FTI was 150 g per ablation point (maximum 476 g). In total 34 min of fluoroscopy were used. No audible steam pop was observed during the applications. No complications occurred. The patient remains free of symptoms at 6 months of follow-up.

## Discussion

### Importance of contact force

Previous research demonstrated that CF is a major determinant for lesion formation and has a greater effect on lesion size than RF output power [10]. CF appears to be associated with FTI, lesion size, and procedural success [10, 11, 14]. Research on pulmonary vein isolation for atrial fibrillation (AF) revealed that the rate of recurrence was associated with the percentage of ablations with very low CFs (<10 g) and predicts recurrence at 12 months. All AF patients who were treated with an average CF of <10 g experienced recurrences, whereas 80 % of the patients treated with a CF of >20 g experienced no AF recurrences [15]. These data demonstrate that proper catheter-tissue contact is crucial to establish appropriate lesions and avoid conduction recovery [13, 15, 16]. Previously only surrogate information regarding CF was available during ablation procedures such as tactile feedback, movement of the catheter tip on fluoroscopy, ST elevation in the unipolar electrogram and impedance monitoring [17, 18]. However, these criteria seemed to be inadequate for estimating real-time CF. [17, 18] Kuck et al. [14] demonstrated that tactile feedback during mapping and ablation is not a reliable parameter and dangerous forces could be applied during catheter manipulation. Furthermore, Kumar et al. [18] found that impedance fall with ablation only has modest predictive value for CF and FTI, and could not accurately differentiate between low and high CFs. Currently, catheters are available that provide direct and continuous feedback regarding CF. The use of this type of catheter has been associated with a decrease in the number of RF applications, which does not result in lesion formation because of insufficient CF [5].

Another important issue during catheter ablation is intermittent CF due to the continuous movements of the heart and respiratory movements. This may occur if the catheter is not in constant contact with the myocardium. This leads to impaired energy delivery and subsequently inferior lesion formation [15]. Reddy et al. showed a strong correlation between low CF and intermittent catheter-tissue contact. Continuous CF feedback allows the operator to adapt the CF during applications, therefore improving lesion quality. However, in some regions it is difficult to achieve a good and stable catheter position with sufficient CF, as we experienced as well during our ablation procedures. Shah et al. [11] demonstrated that FTI is strongly and linearly correlated with lesion depth and volume. Therefore, the operator could compensate low CF by varying RF power and duration of an application.

### Specific concerns during epicardial ablation

Circumstances for catheter ablation on the epicardium are substantially different from endocardial ablation. The absence of a heat sink effect (no circulating blood) results in larger lesions on the epicardium, even though the FTI is twice as low compared with endocardial applications [5]. In the endocardium a CF <10 g or FTI <500 g would lead to non-identifiable lesions on the myocardium. The results from Sacher et al. demonstrate that this was not the case for the epicardium. Wong et al. [6] report that for each doubling of tissue contact between 5 g and 40 g of CF, there is a corresponding doubling in absolute lesion formation and point out the advantage of CF sensing during the ablation. Nevertheless, on the epicardium sufficient lesion formation will occur at lower CF and power output than endocardially. Furthermore, the parallel catheter orientation with lower axial CF compared with endocardial ablation creates more broad and shallow lesions [19]. Using an epicardial approach, complete transmural lesions could be created in the right ventricle (because of the thinner myocardium) [6]. However, for ablation at sites with a thicker myocardial wall a combined endo-epicardial approach could be necessary to achieve transmural lesions.

During ablation, an obstacle for the energy transmission to the epicardium is often the presence of epicardial fat. The low electrical and thermal conductive properties reduce the transmission of energy to the underlying tissue. When ablating at sites with a thick layer of adipose tissue, a high CF is required to achieve at least minimal lesion formation [6]. At sites with epicardial fat of >5 mm, epicardial lesion depth was 1–2 mm when high CF ablation was performed. For ablation at these specific sites, the CF-sensing catheter might have benefits in order to create clinically significant lesions. However, further clinical studies are needed to investigate this issue.

### Calibration of catheter

During endocardial procedures the CF reference (‘no electrode-tissue contact’) is set when the catheter is floating in the heart chamber and can be verified during the procedure. Calibration is thus a crucial part of reliably, or at least relatively, measuring contact force. We describe the first cases in which a non-contact reference in the epicardium was achieved by using an inflated angioplasty balloon (visually beyond the borders of the catheter tip) in an attempt to minimise tissue contact. In this setting the non-contact and subsequent measurements were not considered as absolute values but as an indication of relative force, as the ‘zero’ is set by the operator. In the animal study by Wong et al. [6] normal saline was introduced into the pericardial space to allow catheter floating and therefore zero calibration of CF. However, we assume that the advantages of the angioplasty balloon might be the otherwise compromising haemodynamics. The infusion of saline into the pericardial space could cause tamponade symptoms. This is especially important in patients with a decreased left ventricular function. However, more clinical experience is necessary to evaluate the use of different epicardial calibration methods.

### Safety

Epicardial approach as a first-line treatment of VT has been increasingly used (35.8 %) [7]. Sacher et al. [20] performed a multi-centre study on epicardial VT ablation and demonstrated that major complications could occur during and after the procedure. Although recent techniques are developed to minimise these complications as much as possible, careful evaluation is necessary for the correct approach to ablation, which has been described previously [21–23].

As this paper describes the importance of good CF during ablation for appropriate lesion formation, high CF values could have serious implications regarding procedural safety. Excessive CF values may result in complications such as myocardial perforation, steam pop, cavitation, thrombus formation, oesophageal injury, lung lesions and coronary artery and phrenic nerve damage [5, 13, 24]. It is important that the vector of force is pointing towards the myocardium to prevent pulmonary lesions [5]. As it has been reported that CF information at the epicardial side may not be as useful as endocardially, the CF-sensing catheter could contribute to visualising the vector of the force and therefore energy delivery. Furthermore, Wong et al. [6] described the impact of applied CF and phrenic nerve (PN) injury. Although it is an uncommon complication during epicardial ablation, their results demonstrated that PN injury occurs with increasing, directly applied CF. At 5 or 10 g of CF, PN injury appeared to be unlikely, but was practically universal when 20 g of force was applied.

## Conclusion

We report the first epicardial CF-guided VT ablation in humans with the use of an angioplasty balloon for CF calibration. The information provided on CF could be useful to create more accurate electroanatomical maps, produce better transmural lesions and avoid inefficient ablation points. Although our limited initial experience was successful and safe, more clinical evidence is required to demonstrate the efficacy and safety of CF-guided epicardial VT ablation.
